# The Value of Vengeance and the Demand for Deterrence

**DOI:** 10.1037/xge0000018

**Published:** 2014-10-06

**Authors:** Molly J. Crockett, Yagiz Özdemir, Ernst Fehr

**Affiliations:** 1 Laboratory for Social and Neural Systems Research, Department of Economics, University of Zurich, and Department of Experimental Psychology, University of Oxford; 2 Laboratory for Social and Neural Systems Research, Department of Economics, University of Zurich

**Keywords:** punishment, retribution, deterrence, retaliation, social norms

## Abstract

Humans will incur costs to punish others who violate social norms. Theories of justice highlight 2 motives for punishment: a forward-looking deterrence of future norm violations and a backward-looking retributive desire to harm. Previous studies of costly punishment have not isolated how much people are willing to pay for retribution alone, because typically punishment both inflicts damage (satisfying the retributive motive) and communicates a norm violation (satisfying the deterrence motive). Here, we isolated retributive motives by examining how much people will invest in punishment when the punished individual will never learn about the punishment. Such “hidden” punishment cannot deter future norm violations but was nevertheless frequently used by both 2nd-party victims and 3rd-party observers of norm violations, indicating that retributive motives drive punishment decisions independently from deterrence goals. While self-reports of deterrence motives correlated with deterrence-related punishment behavior, self-reports of retributive motives did not correlate with retributive punishment behavior. Our findings reveal a preference for pure retribution that can lead to punishment without any social benefits.

Punishment of social norm violations is widespread across human societies (Henrich et al., 2006). Under certain conditions, punishment can prevent free-riding and promote cooperation, and many people are willing to “altruistically” punish anonymous strangers, even when it is costly and yields no material or reputational benefits (Fehr & Fischbacher, 2003). Yet the motivational basis of costly punishment is not fully understood. Theories of justice highlight two major proximate motives for punishment: *deterrence* and *retribution* (Bentham, 1789/2000; Kant, 2011). People motivated by deterrence employ punishment to prevent norm violators from repeating their bad behavior in the future; the goal of punishment is to teach a lesson by communicating that a norm has been violated. In contrast, people motivated by retribution employ punishment to cause norm violators to suffer; the goal of punishment is to inflict damage. Although these motives are separate in principle, they are intertwined in practice: any punishment that is communicated to the punisher satisfies both deterrence and retribution goals because it communicates a norm violation and the existence of people who are willing to punish (both of which may reduce future norm violations), and it inflicts damage to the norm violator (satisfying the retributive goal).

Understanding the extent to which punishment is driven by retributive motives has potentially important implications for the design of public institutions to promote social norms. If individuals derive private satisfaction from punishment irrespective of its ability to deter future harms, they may utilize punishment inefficiently in terms of promoting social welfare by, for instance, persisting in punishment even in cases where its future benefits are limited.

Previous studies of punishment motives are consistent with the view that people are concerned about both deterrence and retribution. When asked to provide justifications for punishment, people frequently report a motivation to deter future crimes (Ellsworth & Ross, 1983; Vidmar & Miller, 1980). In hypothetical scenarios, punishment decisions are more sensitive to factors that are primarily associated with retribution (e.g., the severity of the crime) than to factors associated with deterrence (e.g., the likelihood of future transgressions; Carlsmith, 2006; Carlsmith, Darley, & Robinson, 2002). This work provides evidence that both retribution and deterrence motives may play a role in punishment decisions, but based on these studies it remains unclear to what extent people are willing to invest their own resources in punishment that fulfills retribution versus deterrence goals.

Recent studies of costly punishment have demonstrated that people are indeed willing to sacrifice personal payoffs in order to reduce the payoffs of norm violators (Fehr & Fischbacher, 2003). However, these studies have not disentangled the communication of norms and the infliction of damage. It therefore remains unknown to what extent humans will invest their own resources to deter future norm violations versus to exact retribution. In other words, behavioral evidence for costly pure retribution in humans is lacking: it is not known whether individuals are willing to bear the cost of purely retributive sanctions. There is some evidence hinting that people may be willing to pay for retribution alone; punishment levels are substantial even when the implementation of punishment is delayed until after all interaction is over (Fudenberg & Pathak, 2010) and in one-shot games when there is no opportunity for future interactions in the laboratory (Fehr & Fischbacher, 2003). However, the potential effects of punishment on future behavior may well extend beyond the specific context of the laboratory: Subjects who are informed that they are punished for a norm violation in a lab experiment may reduce future norm violations in similar situations outside the lab. Finally, neuroimaging studies have demonstrated activity in reward circuitry, including the striatum and medial prefrontal cortex (mPFC), during punishment of norm violators (Crockett et al., 2013; de Quervain et al., 2004), consistent with the notion that humans derive pleasure from punishment. But since the striatum and mPFC are known to be involved in anticipating distant rewarding outcomes (Kable & Glimcher, 2007), as well as encoding immediately rewarding outcomes (Haber & Knutson, 2010), these studies cannot rule out the possibility that punishment-related responses in these regions reflect the expected social benefits of deterring future norm violations. Moreover, striatal responses during punishment do not necessarily indicate feelings of pleasure (Poldrack, 2006), as the striatum is sometimes also involved in processing aversive outcomes (Delgado, Li, Schiller, & Phelps, 2008).

An additional question concerns differences in punishment motives between second parties who are affected by the norm violation and unaffected third parties. Empirical evidence shows that second parties punish more strongly than unaffected third parties (Fehr & Fischbacher, 2004), and the prevalence of third-party norm enforcement institutions such as juries concords with the common notion that third parties ought to punish in a more impartial or normative manner (Tunick, 1992; von Hirsch, 1986). However, the extent to which retributive motives differ between second- and third-party punishment remains unclear. Comparing second- and third-party punishment is not straightforward, however. Previous attempts suffer from an obvious confound: in second-party punishment only two players are involved, whereas in third-party punishment three players are involved. This is potentially problematic because punishment decisions are sensitive to the presence of an audience (Kurzban, DeScioli, & O’Brien, 2007; Piazza & Bering, 2008). We addressed this issue by examining both second- and third-party punishment in a three-player setting. Our goal in the current study was to characterize the extent of proximate motives for retribution and deterrence in second- and third-party punishment.

## Method

### Participants

Two hundred fifty-nine healthy volunteers provided informed consent and participated in the study, which was approved by the ethics committee of the Department of Economics, University of Zürich. One hundred eleven healthy male volunteers (mean age: 23.2 years) participated in the role of player P, whose behavior was the main focus of the current study. These participants attended testing sessions in the Economics Laboratory at the University of Zürich, for which they received a participation fee of CHF 25 (US$25), plus an additional payment based on their decisions in the study.

### Procedure

#### Three-player trust game with punishment

In our basic setting, three players (a punisher, P; a bystander, B; and a trustee, T) interact anonymously with each other. The punisher (P) and the bystander (B) each receive an endowment of CHF 5. The game has three stages. In the *trust* stage, P and B can entrust their endowment to T. Each entrusted endowment is multiplied by a multiplier *m* and transferred to T. Trustees were instructed that the multiplier could be any integer value between 2 and 6.

**Figure 1 fig1:**
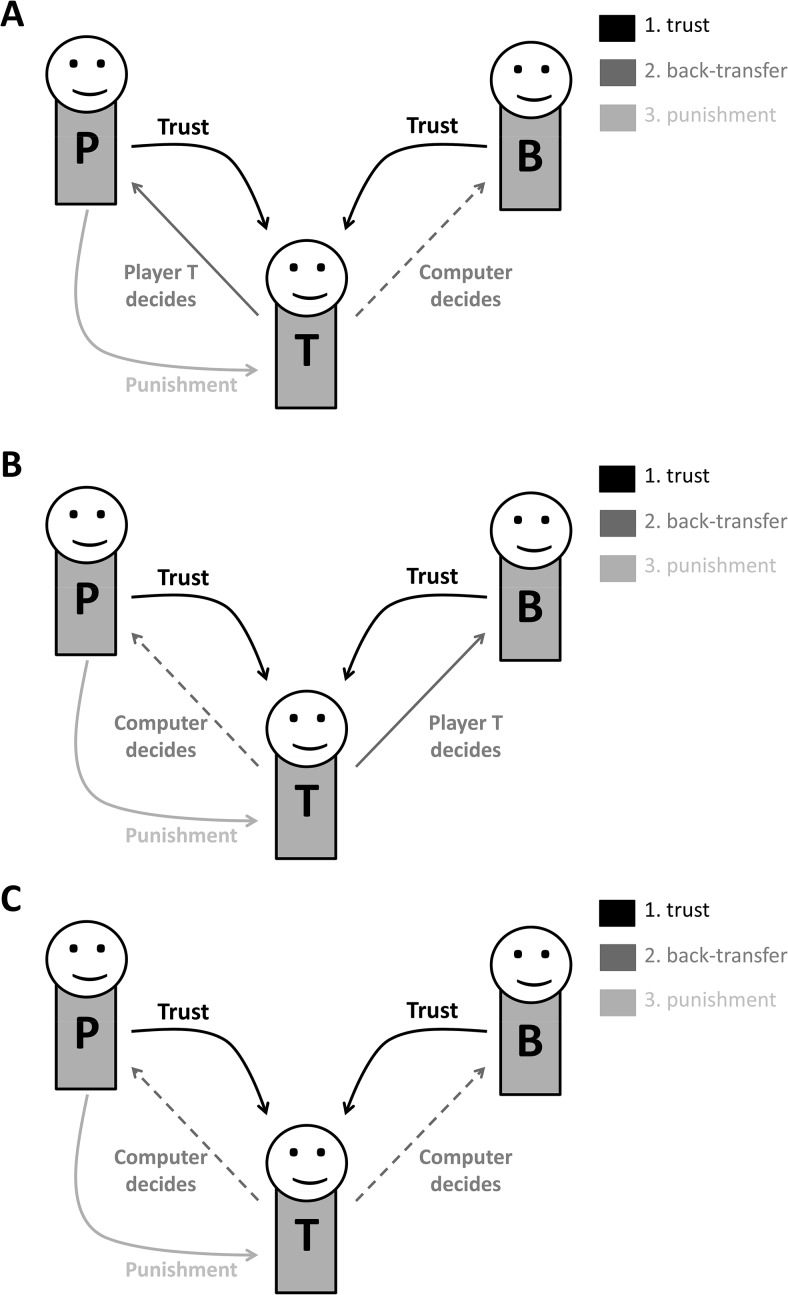
Experimental design. Each trial consisted of three stages. In the *trust* stage, the punisher (P) and bystander (B) entrust their endowments to the trustee (T). In the *back-transfer* stage, P and B receive back-transfers from T. In the *punishment* stage, P decides whether to punish T. We varied the back-transfer mechanism across three experimental conditions. Panel A: In second-party punishment trials, T decides how much to send back to P, while the computer decides how much T sends back to B. Thus, P’s punishment decision concerns T’s intentional back-transfer toward P. Panel B: In third-party punishment trials, T decides how much to send back to B, while the computer decides how much T sends back to P. Thus, P’s punishment decision concerns T’s intentional back-transfer toward B. Panel C: In computer control trials, the computer decides how much T sends back to both P and B. Thus, P’s punishment decision concerns only the payoff differences between players.

In the *back-transfer* stage, T decides what proportion (0%, 25%, or 50%) of the received endowment (CHF 5 * m) to send back to *one* of the players, either P (second-party punishment; see Figure 1A) or B (third-party punishment; see Figure 1B). For the remaining player, the computer decides T’s back-transfer. Thus, in the second-party punishment condition, T decides how to repay P’s trust, while the computer determines how T repays B’s trust. In the third-party punishment condition, T decides how to repay B’s trust, while the computer determines how T repays P’s trust.[Fig-anchor fig1]

Finally, in the *punishment* stage, P receives an additional endowment of CHF 5 and is able to spend up to his entire endowment to reduce T’s payoff; each CHF 0.10 spent by P results in a payoff reduction of CHF 0.20 for T (see the online supplemental materials for details).

In sequential trust and social dilemma games, a strong norm of conditional cooperation applies (Fehr & Fischbacher, 2003, 2004). This norm demands that T respond kindly to initial cooperative acts of P and B in the first stage. Intentional back-transfers of 0% in the second stage therefore unambiguously violate this norm. In the second-party condition only P is the victim of such a norm violation, while in the third-party condition only B is the victim. We therefore expected P to punish T for intentional back-transfers of 0%, since these represent norm violations.

#### Isolating retributive motives

We isolated retributive motives by tightly controlling T’s knowledge of whether he has been punished across two key experimental conditions. Although T’s payoff is always reduced when P punishes him (and P knows this), whether T *learns* about the punishment varies across conditions. In the *open* punishment condition, T receives a written message informing him whether P has punished him. In the *hidden* punishment condition, T is *not* informed whether P has punished him. This was made explicit to the P players in the experimental instructions, and P players had to pass a comprehension quiz to demonstrate their understanding of this before they started the decision-making phase of the experiment.

We were able to control T’s knowledge about his punishment in several ways. T was not informed about either the size of the total endowment that he received through P’s and B’s transfers or the size of the back-transfer determined by the random device. Moreover, because a specific final payoff in the technically possible payoff range could arise in many different ways, the final payoff also provided no information about punishment (see the supplemental materials for a detailed explanation).

We used detailed instructions to ensure that the punisher P was aware of the difference between open and hidden punishment when he made his punishment decisions. We confirmed this with a comprehension quiz (see the online supplemental materials for details).

Our experimental design provides a stringent test for the existence of retributive motives in humans. The hidden punishment condition excludes the deterrence motive, because deterring future norm violations requires that the perpetrator know that he has been punished. Thus, higher punishment of unfair back-transfers (relative to fair back-transfers) in the hidden condition reflects retributive motives (i.e., the private satisfaction derived from reducing the payoff of a norm violator). In contrast, higher punishment of unfair back-transfers (relative to fair back-transfers) in the open condition reflects a combination of retribution and deterrence motives (see Table 1). Because the open condition has the same retributive effects as the hidden condition, but with the added benefit of deterrence, we expected open punishment of unfair back-transfers to be both more likely and more substantial than hidden punishment of unfair back-transfers. And based on previous studies suggesting a potential role of retributive motives in punishment (Carlsmith, 2006; Carlsmith et al., 2002), we expected to observe higher punishment of unfair back-transfers (relative to fair back-transfers) in the hidden condition, despite the fact that unambiguous behavioral evidence for pure retribution is currently lacking.[Table-anchor tbl1]

#### Controlling for payoff-based motives

Decisions to punish can also be motivated by inequality aversion (Fehr & Schmidt, 1999) or other types of payoff-based social preferences such as spite (Jensen, 2010). People who dislike inequality will punish others with higher payoffs, regardless of whether the target of punishment is not responsible for payoff allocations (Blount, 1995; Falk, Fehr, & Fischbacher, 2008). Likewise, spiteful subjects punish regardless of whether the trustee decided intentionally or whether a random device determined the back-transfer (see Table 1). To separately control for such motives, we implemented a “computer control” condition in which T’s back-transfer decisions vis-à-vis both P and B were unintentional (i.e., determined by the computer; see Figure 1C). In the computer control condition, punishers faced a set of decisions that were identical to those in the two experimental conditions in all respects aside from the intentionality of the trustee T (see Figure S1 in the online supplemental materials).

#### General procedure

We collected the decisions of B and T players in advance (see the online supplemental materials), so that we were able to face each player P with an identical set of games without using deception. Each punisher P played a series of 54 anonymous one-shot trust games with punishment, each with different individuals in the roles of B and T. Each player P faced the same set of 54 games, reflecting a factorial within-subject design that crossed (a) level of T’s back-transfer (0%, 25%, or 50%), (b) second- versus third-party punishment, (c) whether punishment was open or hidden, and (d) whether T’s back-transfer was intentional or unintentional (see Figure S1 in the online supplemental materials). The dependent measure was the amount P spent on punishment in each game. Subjects had unlimited time to make their punishment decisions. Punishment decision data were analyzed in SPSS 18 using the generalized estimating equations procedure, which generates for each tested main effect and interaction a chi-square statistic, a 95% confidence interval, and an associated *p* value. We used an independent working correlation matrix given that participants played one-shot games and thus the correlation between repeated measurements should be low. For analysis of binary (yes/no) punishment decisions, we used a logistic link function, and for analysis of continuous punishment amounts, we used a linear link function. Effect sizes were computed using Cohen’s *d*.

Following the 54 games, participants completed a questionnaire concerning their motivations for punishment (see the supplemental materials). Both the games and the questionnaire were implemented using z-Tree (Fischbacher, 2007). At the end of the session, one of the 54 games was randomly selected for payment for each subject. Subjects in the role of P received their payments in cash immediately. Subjects in the roles of T and B whose decisions were implemented in the randomly selected game received their payments by mail. If the randomly selected game was one with open punishment, the payment sent to T included a letter that revealed whether P punished T and by how much.

## Results

### Retribution and Deterrence in Second-Party Punishment

**Figure 2 fig2:**
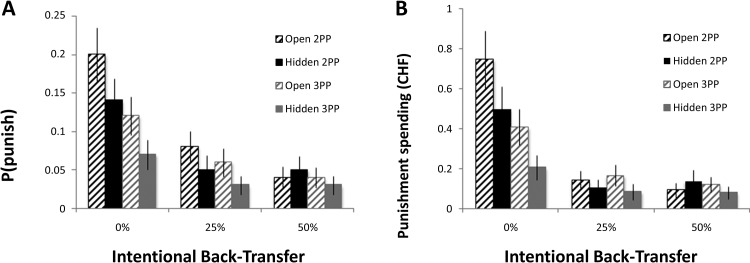
Retribution and deterrence in second- and third-party punishment. Punishment likelihoods (Panel A) and mean amount spent (Panel B) for second-party punishment (2PP; black) and third-party punishment (3PP; gray), in the open (lined) and hidden (solid) conditions. Error bars depict the standard error of the mean. CHF = Swiss franc.

In second-party punishment trials, P decided whether and how much to punish T for intentionally sending back 0%, 25%, or 50% of the money to P. As expected, back-transfer level had a significant effect on second-party punishment (likelihood: χ^2^ = 16.781, *p* < .001, *d* = 0.84; amount: χ^2^ = 19.663, *p* < .001, *d* = 0.93); P was much more likely to punish and spent more to punish T when he sent back 0% of the money, relative to 25% and 50%. Critically, subjects distinguished between fair and unfair back-transfers in both the open condition (likelihood: χ^2^ = 24.907, *p* < .001, *d* = 1.08; amount: χ^2^ = 21.673, *p* < .001, *d* = 0.99; see Figure 2, striped black bars) and the hidden condition (likelihood: χ^2^ = 9.544, *p* = .008, *d* = 0.61; amount: χ^2^ = 13.419, *p* = .001, *d* = 0.74; see Figure 2, solid black bars). The latter result provides unambiguous evidence for second-party retributive motives in humans. Finally, in line with our predictions, open punishment was both more likely and more substantial than hidden punishment, particularly for 0% back-transfers (Open × Back-Transfer interaction, likelihood: χ^2^ = 12.487, *p* = .002, *d* = 0.71; amount: χ^2^ = 11.419, *p* = .003, *d* = 0.68). These findings demonstrate that the preference to communicate norms through punishment also plays an important role for punishment decisions.[Fig-anchor fig2]

### Retribution and Deterrence in Third-Party Punishment

In third-party punishment trials, P decided whether and how much to punish T for intentionally sending back 0%, 25%, or 50% of the money entrusted to him by B. We found that participants were less likely to engage in third-party punishment than second-party punishment (χ^2^ = 15.501, *p* < .001, *d* = 0.81) and spent less on third-party punishment than second-party punishment (χ^2^ = 10.505, *p* = .001, *d* = 0.65). Thus, third-party punishment is less likely and less strong even when controlling for the number of players involved in the interaction.

To what extent did retribution motivate third-party punishment? Similar to second-party punishment, we observed a main effect of T’s back-transfer to B on P’s decisions to punish T (likelihood: χ^2^ = 16.049, *p* < .001, *d* = 0.82; amount spent: χ^2^ = 12.856, *p* < .001, *d* = 0.72). Again, subjects distinguished between fair and unfair back-transfers in both the open condition (likelihood: χ^2^ = 18.266, *p* < .001, *d* = 0.89; amount: χ^2^ = 12.019, *p* = .002, *d* = 0.70; see Figure 2, striped gray bars) and the hidden condition (likelihood: χ^2^ = 8.122, *p* = .017, *d* = 0.56; amount: χ^2^ = 5.909, *p* = .052, *d* = 0.47; see Figure 2, solid gray bars), providing evidence for third-party retributive motives. Finally, as was the case for second-party punishment, open punishment was both more likely and more substantial than hidden punishment, across all levels of back-transfer (main effect of open, likelihood: χ^2^ = 5.542, *p* = .019, *d* = 0.46; amount: χ^2^ = 10.915, *p* = .002, *d* = 0.66). The effect of norm communication on punishment of unfair back-transfers was no larger for third-party punishment than for second-party punishment (Party × Open × Back-Transfer interaction, likelihood: χ^2^ = 0.613, *p* = .736, *d* = 0.149; amount: χ^2^ = 2.211, *p* = .331, *d* = 0.285).

### Controlling for Payoff-Based Motives

One potential alternative explanation for the observation of hidden punishment is that such punishment reflects inequality aversion, spite, or other types of purely payoff-based social preferences rather than retributive motives. Note that retributive motives can only play a role when back-transfers are intentional, while the punisher’s payoff-based social preferences might play a role in the punishment of both intentional and unintentional back-transfers. Therefore, we can rule out these alternative explanations by comparing hidden punishment of intentional back-transfers by T with hidden punishment of unintentional back-transfers by T (matched for amount). In computer control trials (see Figure 1C), the computer decided player T’s back-transfers to both P and B; therefore, in these trials, player T was not responsible for the level of back-transfer. Thus, the observation of higher punishment in the hidden-intentional condition, relative to the hidden-unintentional condition, constitutes evidence for retributive motives over and above purely payoff-based social preferences.

**Figure 3 fig3:**
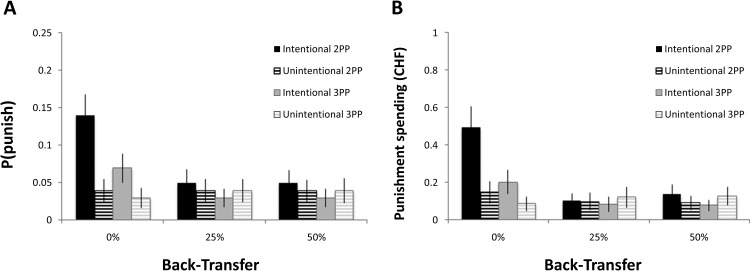
Retribution and payoff-based motives in second- and third-party punishment. Punishment likelihoods (Panel A) and mean amount spent (Panel B) for hidden punishment levels when back-transfers resulted from intentional decisions by trustees (solid) versus when back-transfers resulted from the computer’s decision (lined), in the second-party punishment (2PP; black) and third-party punishment (3PP; gray) conditions. Error bars depict the standard error of the mean. CHF = Swiss franc.

We observed significantly more punishment in the hidden-intentional condition, relative to the hidden-unintentional condition. For second-party hidden punishment, there was a significant main effect of intentionality on punishment (likelihood: χ^2^ = 9.875, *p* = .002, *d* = 0.62; amount: χ^2^ = 10.125, *p* = .001, *d* = 0.63; see Figure 3, black bars); intentional back-transfers were punished more strongly than unintentional ones of equal value. This effect of intentionality was strongest for 0% back-transfers, as evidenced by a significant interaction between intentionality and back-transfer (likelihood: χ^2^ = 7.217, *p* = .027, *d* = 0.53; amount: χ^2^ = 9.525, *p* = .009, *d* = 0.61). For third-party hidden punishment, there was also a significant interaction between intentionality and back-transfer; intentional back-transfers were punished more strongly than unintentional back-transfers, but only for the most unfair (0%) back-transfers (likelihood: χ^2^ = 6.950, *p* = .031, *d* = 0.52; amount: χ^2^ = 6.732, *p* = .035, *d* = 0.51; see Figure 3, gray bars). Thus, payoff-based motives could not completely explain hidden punishment in either second- or third-party punishment.[Fig-anchor fig3]

We next examined differences in retributive motives between second- and third-party punishment, focusing exclusively on trials in the hidden condition. The average level of hidden punishment of *unintentional* 0% transfers did not differ significantly between second- and third-party conditions (χ^2^ = 1.736, *p* = .188, *d* = 0.252), suggesting that second- and third-party punishment were matched in terms of purely payoff-based social preferences. However, the average amount of hidden punishment of *intentional* 0% transfers was significantly greater in second- than third-party punishment (χ^2^ = 7.125, *p* = .008, *d* = 0.52). This observation was confirmed by a significant two-way interaction between party and intentionality (χ^2^ = 4.558, *p* = .033, *d* = 0.41) within the hidden condition; punishment in the hidden-intentional condition, *relative to* the hidden-unintentional condition, was greater in second-party than in third-party punishment. These results suggest that retributive motives, while present in both second- and third-party punishment, are stronger in the former than in the latter.

### Self-Reported Motives for Retribution and Deterrence

We next explored the correspondence between subjects’ self-reported motives for punishment and their actual punishment behavior. After they had made all their decisions, we asked subjects to indicate on a Likert scale the extent to which their punishment decisions were motivated by factors associated with deterrence and factors associated with retribution (see the supplemental materials for details of the factor analysis). Endorsement of retributive motives was low, with a mean rating of 1.75 (*SE* = 0.13) on a 5-point scale. Endorsement of deterrence motives was significantly higher (*M* = 3.06, *SE* = 0.20), *t*(110) = 6.769, *p* < .001, *d* = 1.29). We then correlated subjects’ self-reported ratings against their own behavior. Our behavioral measure of deterrence motives—the difference between amount spent on open relative to hidden punishment of unfair (0%) back-transfers—was positively correlated with self-reported deterrence motives (*r* = .417, *p* = .004, *d* = 0.92). However, our behavioral measure of retributive motives—the amount spent on hidden punishment of intentional relative to unintentional unfair (0%) back-transfers—was not significantly correlated with self-reported retributive motives (*r* = .017, *p* = .913). The relationship between self-report and behavior was stronger for deterrence motives than for retributive motives (*Z* = 1.96, *p* = .05, *d* = 0.38). In fact, self-reported retributive motives did not significantly predict any aspect of punishment behavior (all *p*s > .687).

## Discussion

Our findings provide unambiguous behavioral evidence that people are willing to invest personal resources in pure retribution without the possibility of deterrence. We observed higher punishment of unfair back-transfers than fair back-transfers even in our hidden treatment, where the norm-enforcing properties of punishment were completely removed. Retributive punishment was evident in both second- and third-party punishment settings and could not be completely explained by inequality aversion or other purely payoff-based preferences such as spite. These results indicate that people value reducing the payoffs of norm violators, even in the absence of any potential future social benefits of punishment.

At the same time, our data suggest that punishers derive additional value from the opportunity to communicate norms. Costly punishment was both more likely and more substantial when the target of punishment would learn that he was punished, controlling for material damage. This finding is consistent with previous work showing that the opportunity to communicate norms (sometimes called *emotion expression*) can serve as a substitute for inflicting material damage (Xiao & Houser, 2005; Yamagishi et al., 2009).

Alternatively, it is possible that the communication of norms is driven to some extent by a retributive desire to inflict emotional damage (in addition to material damage). Some evidence has suggested this is indeed the case. Dictators who anticipate receiving a written message from their recipient give significantly higher amounts than do those who will not receive a message, indicating that non-material sanctions carry emotional weight (Ellingsen & Johannesson, 2008; Xiao & Houser, 2009). It is therefore possible that the present study underestimated the extent to which retributive motives drive costly punishment.

We provide a novel method for directly comparing second- and third-party punishment within a single setting. Holding constant the number of players involved in the interaction, the payoff of the punisher, and the relative payoffs between the punisher and the other players, we observed stronger second-party punishment than third-party punishment. Preferences for the communication of norms did not significantly differ between second- and third-party punishment. However, retributive motives were stronger in second- than third-party punishment. This suggests that personal suffering amplifies the demand for retribution but not the communication of norms.

Notably, subjects’ distinction between open and hidden punishment was strongest for the unfair back-transfers. We observed a few instances of *antisocial* punishment of fair 50% back-transfers (Gächter & Herrmann, 2009; Herrmann, Thöni, & Gächter, 2008; Rand, Armao, Nakamaru, & Ohtsuki, 2010; Rand & Nowak, 2011); unlike punishment of unfair back-transfers, the amount of antisocial punishment did not differ between open and hidden conditions. This suggests that antisocial punishment is driven by a desire to inflict damage on fair players, rather than a desire to communicate a norm of non-cooperation. This hypothesis could be tested further using methods similar to those use in the present study, but in populations with higher occurrences of antisocial punishment (Herrmann et al., 2008).

Our methods also enabled us to disentangle punishment motives within-subject. Previous research on costly punishment behavior has not explicitly separated preferences about material payoffs from preferences about the communication of norm violations, since the target of punishment was always informed that he has been punished. Here we were able to measure the relative contributions of both types of preferences to punishment behavior and to compare behavioral preferences with self-reported motives. Such comparisons can be valuable because people may lack insight into their own motives (Nisbett & Wilson, 1977) or be reluctant to disclose motivations that are less socially desirable. Consistent with this view, in our study subjects rarely endorsed retributive motives in the self-report questionnaire. Meanwhile, subjects were more likely to endorse motives for deterrence, perhaps because such motives are more socially desirable. Self-reported motives for deterrence were significantly correlated with our behavioral measure of deterrence, but self-reported motives for retribution were not correlated with our behavioral measure of retribution, or indeed any aspect of punishment behavior. Further research is needed to understand the factors that moderate the correspondence between self-reported motives and behavior.

An intriguing open question is whether preferences for retribution versus deterrence depend on distinct neural systems. Punishment decisions engage brain regions involved in the computation of value, including the striatum and mPFC (Baumgartner, Knoch, Hotz, Eisenegger, & Fehr, 2011; Crockett et al., 2013; de Quervain et al., 2004), but also regions involved in forward planning and goal-directed decision making, including the dorsolateral prefrontal cortex (Baumgartner et al., 2011; Buckholtz et al., 2008; Sanfey, Rilling, Aronson, Nystrom, & Cohen, 2003). While activity in the striatum tracks the amount of material damage inflicted by punishment (de Quervain et al., 2004), prefrontal regions may be sensitive to whether punishment is likely to deter future harms (Buckholtz et al., 2008; Buckholtz & Marois, 2012). Environmental factors such as stress are known to disrupt prefrontal function (Robbins & Arnsten, 2009) and may therefore alter the nature of punishment decisions. Understanding the influence of the environment on punishment decisions has important implications for the criminal justice system (Danziger, Levav, & Avnaim-Pesso, 2011).

The National Council on Crime and Delinquency has declared that sentencing should not be based on revenge and retribution (Tunick, 1992; von Hirsch, 1986). This view is consistent with our finding that retributive motives were less forceful in third-party punishment, relative to second-party punishment. However, our findings also cast some doubt on the notion that “impartial observers” are capable of meting out punishments in a normative manner immune to emotional influences; retributive motives still explained a substantial portion of third-party punishment. This is perhaps not so surprising in light of humans’ remarkable capacity for empathy. Observing harm to another engages similar brain regions as those that signal harm to the self (Singer et al., 2004). Thus, if the desire for retribution arises in response to self-directed harm, it may be similarly triggered by harm against others, to the extent that harm against others feels aversive (Batson et al., 2007). Since empathy is stronger for ingroup members, retributive motives may play a stronger role in third-party punishment when the victim is an ingroup member (Lieberman & Linke, 2007). This insight has potential implications for determining the composition of juries.

Research in evolutionary game theory has examined how punishment might have evolved (Boyd, Gintis, Bowles, & Richerson, 2003; Rand et al., 2010). In most of these models, the effects of punishment operate by reducing the fitness of non-cooperators, thus making them less plentiful in subsequent generations, rather than by reforming the behavior of non-cooperators in the current generation. These models therefore assume that one key function of punishment is to make non-cooperators worse off, which does not require their knowledge that they have been punished—akin to our hidden-punishment condition. Our finding that people are indeed willing to punish non-cooperators even when such punishment cannot serve a deterrent function thus lends psychological support to the punishment mechanism employed by evolutionary models.

Although costly punishment often has the effect of increasing cooperation (Balliet, Mulder, & Van Lange, 2011; Fehr & Fischbacher, 2003), whether people punish “altruistically” in a psychological sense, with the explicit goal of promoting cooperation, remains hotly debated (Guala, 2012; McCullough, Kurzban, & Tabak, 2013; Yamagishi, Horita, & Mifune, 2012). Our results offer some resolution to this debate. We show that punishers are motivated in large part by a genuine preference to reduce the payoffs of norm violators, even in the absence of opportunities to enforce norms. Such hidden punishment cannot be considered altruistic, because it cannot produce any social benefits. At the same time, we provide evidence that punishers have preferences for norm enforcement, in that punishers are more likely to punish, and spend more on punishment, when norms can be communicated. This could reflect an altruistic motive to deter future norm violations or may instead reflect a retributive desire to inflict emotional harm. Regardless, the substantial contribution of retributive motives to costly punishment suggests that informal peer sanctions may not be the most efficient means of promoting cooperation. Humans possess psychological mechanisms that can lead to destructive behavior that is sub-optimal in terms of deterring future harms. Further research is needed to understand how such motives influence the decisions of judges and juries.

## Supplementary Material

10.1037/xge0000018.supp

## Figures and Tables

**Table 1 tbl1:** Different Punishment Motives Predict Different Patterns of Punishment Across Experimental Conditions

Punishment motive	Prediction
Deterrence	Open Unfair > Fair
	Hidden Unfair = Fair
	Computer Unfair = Fair
Retribution	Open Unfair > Fair
	Hidden Unfair > Fair
	Computer Unfair = Fair
Payoff-based (e.g., spite, inequality aversion)	Open Unfair > Fair
	Hidden Unfair > Fair
	Computer Unfair > Fair
