# Supplementary material for: The Value of Vengeance and the Demand for Deterrence
Source: J Exp Psychol Gen. 2014 Oct 6;143(6):2279–86. doi: 10.1037/xge0000018 (PMC4242077; doi:10.1037/xge0000018)
Supplement: Supplementary file 1 [file xge.Crockett.SUPP.FINAL.docx]

**Supplemental Materials**

**The Value of Vengeance and the Demand for Deterrence**

**By M. J. Crockett et al., 2014, *Journal of Experimental Psychology: General***

**http://dx.doi.org/10.1037/xge0000018**

**Experimental Procedure**

All interactions in the experiment were fully anonymous. We collected the decisions of participants in the role of T and B in advance of the main experiment. These decisions were collected at the end of other experimental sessions in the Economics Laboratory by paper-and-pencil. These participants were instructed that their decisions could be matched with future participants in the role of player P, and that they would receive the money resulting from the subsequent use of their decisions via post. We employed this procedure so that we could present participants in the role of player P with a set of decisions that displayed specific parameters, but without using deception. All aspects of the procedure were fully transparent to all participants (i.e., no deception was used).

In the main experiment, participants in the role of player P first read a set of detailed instructions and completed a comprehension quiz, which had to be passed successfully in order to continue with the experiment. Importantly, to pass the quiz, P players had to correctly answer questions that indicated whether they understood that in the hidden condition T players could not know whether they had been punished. All participants passed the comprehension quiz.

Next, each participant decided whether to entrust their initial endowment of CHF 5 to all players in the role of T that they would face during the entire experiment. Subjects who entrusted the CHF 5 then played a series of 54 anonymous one-shot trust games with punishment, each with different individuals in the roles of B and T. Since we collected the decisions of B and T players in advance, we were able to face each player P with the same set of 54 parameterizations, reflecting a factorial within-subjects design that crossed (a) T’s back transfer to B (0%, 25%, or 50%), (b) T’s back transfer to P (0%, 25%, or 50%), (c) whether T's intentional decision affected P, B, or neither; and (d) whether punishment was open or hidden (see Figure S1). We only selected B players who decided to entrust the CHF 5 to the trustee T.

In each game, the initial entrusted endowment of CHF 5 was multiplied by 4. This multiplier was known to players P and B but not player T. Next, participants viewed the information about the current trial. Finally, participants received an additional endowment of CHF 5 and decided how much to spend to reduce the payoff of player T. Each CHF 0.10 spent on punishment reduced the payoff of player T by CHF 0.20. Participants had unlimited time to make their decisions.

Player T was informed that the multiplier *m* could lie in the range of 2 to 6. However, *m* was always kept constant at 4 such that Player T was never able to infer whether he has been punished from his final payoff alone.

**Punishment motive questionnaire**

After the decision-making phase, all participants filled out an electronic questionnaire. We examined post-hoc self-reported punishment motives by measuring agreement with the following 7 statements using a 5-point Likert scale:

I reduced the payoff of the punisher …

1. … because it was fun.
2. … to teach him a lesson.
3. … because I wanted him to suffer.
4. … to change his future behavior.
5. … to reduce inequality.
6. … to demonstrate my power.
7. … to take revenge.

With these ratings, we conducted a principal components analysis with varimax rotation and Kaiser normalization. This revealed two independent factors with eigenvalues greater than one, accounting for 66% of the total variance in the ratings. The first factor, *deterrence,* accounted for 35% of the variance and included items 2, 4, and 5. The second factor, *retribution,* accounted for 32% of the variance and included items 1, 3, 6, and 7.

For the correlation analyses reported in the main text, we computed means of the retribution and deterrence items to derive retribution and deterrence scores for each subject. The questionnaire also included items about the quality of the instructions as well as the age and educational level of the participants.


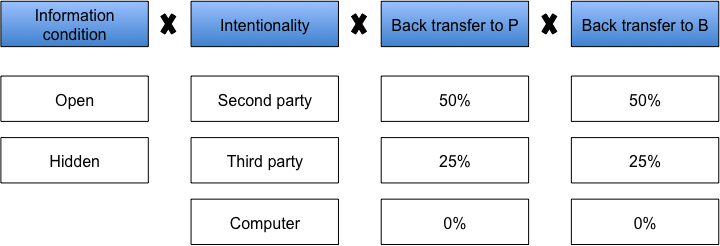


*Figure S1.* Summary of factorial design. Participants in the role of the punisher who entrusted the CHF 5 in the first stage passed through 54 treatments, each a particular combination of the information condition, the intentionality, the back transfer to P, and the back transfer to B.

**Payoff and Information Structure of the Trustee**

By tightly controlling the information available to T, we were able to ensure that in the hidden punishment condition T could not reasonably infer whether he has been punished. A low overall payoff for T could, for example, be due to (i) a low multiplier or (ii) a high computer-driven back transfer or (iii) a certain punishment level. However, because T knows nothing about these three variables, he cannot make any reasonable inferences about the punishment level (see SI for a detailed explanation).

In this section we show that in the hidden condition the Trustee can never infer from his final payoff that he has been punished. First define the following variables:

- Multiplier: m ∈ M = [2, 6]
- Impact of punishment: p ∈ P = [0, 10]
- Intentional back transfer decision of the Trustee: i ∈ I = {0, 0.25, 0.5}
- Back transfer decision of the computer: j ∈ J = {0, 0.25, 0.5}
- Payoff of the Trustee: π = 5m(1 – i) + 5m(1 – j) – p = 5m[2 – (i+j)] – p
- Information set of the Trustee in the hidden condition: S = {i, I, J, M, P, π}

The realization of the multiplier and the back transfer decision of the computer were unknown to the Trustee. Hence, from the perspective of the Trustee each of the three intentional back transfer decisions lead to a different range of possible payoffs:

(1.1) π(i = 0) ∈ [15 – p, 60 – p]

(1.2) π(i = 0.25) ∈ [12.5 – p, 52.5 – p]

(1.3) π(i = 0.5) ∈ [10 – p, 45 – p]

The ranges in (1.1)-(1.3) are calculated by combining the range of the multiplier with the possible back transfer decisions of the computer, holding the intentional back transfer, which is known to the Trustee, constant. The lower bound of each range in (1.1)-(1.3) results from the lowest possible multiplier, m = 2, and the highest possible back transfer decision of the computer, j = 0.5. The upper bound of each range in (1.1)-(1.3) results from the highest possible multiplier, m = 6, and the lowest possible back transfer decision of the computer, j = 0.

Since the multiplier was, however, kept constant at m = 4, the following payoffs might have *actually* occurred, depending on the intentional back transfer decision, the back transfer decision of the computer and the punishment decision^[[1]](#footnote-1)^:

(2.1) π(i + j = 0, m = 4) = 40 – p

(2.2) π(i + j = 0.25, m = 4) = 35 – p

(2.3) π(i + j = 0.5, m = 4) = 30 – p

(2.4) π(i + j = 0.75, m = 4) = 25 – p

(2.5) π(i + j = 1, m = 4) = 20 – p

With a sufficiently low payoff the Trustee could theoretically infer that p was larger than zero if the trustee earns less than the lower bound in (1.1 – 1.3) for p=0. Thus, a positive level of p could only be inferred if the following occurred:

(3.1) π(i = 0) < 15

(3.2) π(i = 0.25) < 12.5 or

(3.3) π(i = 0.5) < 10

If, for example, the Trustee had decided to transfer back i = 0 and had obtained a payoff smaller than 15 it would have been possible to infer that p > 0 based on (1.1). But by (2.3) the lowest possible payoff that might have *actually* occurred, if i = 0, was 30 – p. This follows from the fact that i + j cannot be larger than 0.5, if i = 0. However, since the punishment technology did not allow p to be larger than 10, the actual payoff for i = 0 could have never been lower than 20. Therefore, the actual payoff in this scenario could have never been lower than 15, the threshold value given by (3.1), and thus the Trustee cannot infer that he has been punished, if i = 0.

Now we show that this argument also holds for i = 0.25 and i = 0.5. Table S1 depicts for each combination of i and j the required punishment impact such that the actual payoff given by (2.1)-(2.5) would be lower than the corresponding threshold value given by (3.1)-(3.3). Because in all scenarios p ∈ [0, 10] is too low to sufficiently decrease the actual payoff, the Trustee can never deduce that he has been punished, unless the information is explicitly provided.

Table S1

*Required Impact of Punishment Such That the Trustee Could Infer That He Has Been Punished*


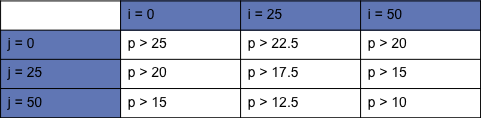


**Appendix 1**

**Instructions and Quiz**

**Instructions**

**Player P instructions**

You are now participating in an experiment which is sponsored by various research foundations. The experiment is completed in cooperation with the Department of Economics at the University of Zurich, Switzerland. Please read the following instructions carefully. If you have any questions, please ask an experimenter.

You will receive a **fixed amount of CHF 25** for participation in this experiment. You can also **earn money in addition** to this fixed amount based on your decisions during the experiment. Upon conclusion of the experiment, you will receive your payment in cash.

These instructions are solely reserved for your private information. Communication is strictly forbidden during the study. If you have any questions, please ask us. Disregarding these rules leads to exclusion from the experiment and from any payments.

The data collected in this experiment will be kept strictly confidential. Future publications only represent average results. Drawing inference on particular participants will not be possible, and your anonymity will be maintained at all times.

There are three types of participants in this experiment, participants A, participants B, and participants C. **You are a Participant A.** Participants B and C have already made their decisions and are, therefore, not present in the lab today. We will further explain to you the background of this procedure in the last section. You will be matched sequentially with a group of 54 different pairs of people who are in the role of participant B and participant C.

You will participate in a three-step experiment with each of the 54 pairs of participant B and participant C. You will interact with each participant only once. Consequently, you will interact with 108 different people in this experiment: 54 participants B, and 54 participants C. The whole experiment will be completely anonymous. Neither will you know the identity of any other participant, nor will any other participant know your identity.

The experiment consists of **three steps**. On the following pages we will explain you these three steps and the exact procedure of the experiment. The payoffs on the following pages are related to those payoffs which you can earn **in addition** to the fixed payment of CHF 25.

**Procedure for the three steps.** You and participant B will each receive an endowment of **CHF 5** at the beginning of the experiment.

Independent from each other, you both must decide in **step one** whether you want to transfer your endowment of CHF 5 to Participant C or if you will transfer nothing.

If you decide to transfer the CHF 5, it will be multiplied by the factor 4, meaning that Participant C will receive a total endowment of CHF 20. So if both you and Participant B transfer the CHF 5 to Participant C, he will receive CHF 20 from you and CHF 20 from Participant B. Participant C knows whether you and Participant B transferred the CHF 5 to him or not. But he is not informed about the factor with which each of the CHF 5 are multiplied. He only knows that the multiplier lies in a range between 2 and 6. Therefore, Participant C does not know the amount which he receives from you and Participant C after the multiplication.

If you decide not to transfer the CHF 5, you will keep the CHF 5 and you will not participate in the following steps of the experiment. If Participant C does not receive any CHF 5 in step one, his payoff for this round will be CHF 0. The following diagram illustrates possible money flows in step one:


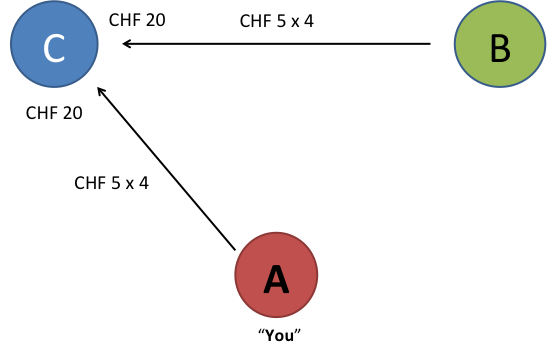


Example for Step 1

There will be two decisions made in **step two**:

1. Participant C will send back to you 0%, 25%, or 50% of what he received from you.
2. Participant C will send back to Participant B 0%, 25%, or 50% of what he received from Participant B.

Sometimes one of these decisions will be made by Participant C, and the other will be made by the computer. At other times, both decisions will be made by the computer. Participant C will never learn the decision made by the computer.

Note that Participant C will make his decisions about the **percentage** of the endowment he will transfer back to you and Participant B. Since he is **not informed** about the factor with which each CHF 5 is multiplied, Participant C **does not know** the size of the total endowment at the time he makes his decision.

In step two you will be informed of participant B’s decision, participant C’s decision, and the decision of the computer. You will thus have full information about the intermediate payoffs of all participants. You do not have to make an active decision in step two.

In **step three,** you receive an additional CHF 5. You can then use this money to reduce the payoff of Participant C. For every CHF 1 you spend, Participant C’s payoff will be reduced by CHF 2.

There are **two types of decisions** you will make in step three: “OPEN” and “HIDDEN.” In the “OPEN” type Participant C will **be informed** of your decision in step three when he receives his payment. In the “HIDDEN” type Participant C will **not be informed** of your decision when he receives his payment. Participant C will never learn the size of the starting endowment or the decision of the computer, so he won’t be able to know whether you chose to reduce his payoff based on the size of his payoff alone. He will only know whether you chose to reduce his payoff on “OPEN” decisions, where he is explicitly informed of your decision.

You will be informed whether your decision is “OPEN” or “HIDDEN” before you make your decision. Participant B is not involved in step three and does not have the opportunity to reduce the payoff of Participant C.

At the end of step three, the final payoffs for all participants will be calculated. After you have participated in the experiments with all 54 pairs of participants B and C, one round will be randomly selected by the computer after you press the random-button. The payoffs from this round will be implemented. You will receive your payoff in cash at the end of the session, and participants B and C will be sent their payoffs by post.

It is important to emphasize that Participant C will **only be able to know your decision in step 3 if he is explicitly informed about it**. We illustrate this point in the example below.

**Example:**

You and Participant B both decide to transfer your CHF 5 to Participant C.

Participant C decides to send back 25% of what he received from you. The computer decides how much percent to send back of what Participant C received from Participant B.

Later, Participant C receives a payment of CHF 30 by post.

Note that this payment could be generated by more than one scenario. For example:

***Scenario 1 from the perspective of participant C.*** You and participant B both decide to transfer your CHF 5 to Participant C. This is multiplied by 4, resulting in a starting endowment for Participant C of CHF 20 from you and CHF 20 from Participant B. However, Participant C does not know these amounts. Participant C decides to send back 25% of what he received from you, resulting in CHF 5 for you and CHF 15 for Participant C. The computer decides to send back 25% of what Participant C received from Participant B, resulting in CHF 15 for Participant C and CHF 5 for Participant B. You receive an additional endowment of CHF 5, and decide not to spend any money to reduce Participant C’s payoff. Therefore the final payoffs for all participants are the following:

You: CHF 10

Participant B: CHF 5

Participant C: CHF 30

***Scenario 2 from the perspective of participant C.*** You and participant B both decide to transfer your CHF 5 to Participant C. This is multiplied by 5, resulting in a starting endowment for Participant C of CHF 25 from you and CHF 25 from Participant B. Remember that the factor lies between 2 and 6 from the view of Participant C! Participant C decides to send back 25% of what he received from you, resulting in CHF 6.25 for you and CHF 18.75 for Participant C. The computer decides to send back 25% of what Participant C received from Participant B, resulting in CHF 18.75 for Participant C and CHF 6.25 for Participant B. You receive an additional endowment of CHF 5, and decide to spend CHF 3.75 to reduce Participant C’s payoff by CHF 7.50. Therefore the final payoffs for all participants are the following:

You: CHF 7.50

Participant B: CHF 6.25

Participant C: CHF 30

Note that these are just two possible scenarios that could generate a final payoff for Participant C of CHF 30. In Scenario 1, you decided not to reduce Participant C’s payoff, but in Scenario 2, you did decide to reduce Participant C’s payoff. Thus, he would have no way of knowing whether you reduced his payoff based on his final earnings alone. **He can only learn your decision if he is explicitly informed about your decision (i.e. in the “OPEN” type)**.

**Procedure of the entire experiment.** You will complete this three step experiment with 54 different pairs of people who are in the role of participant B and participant C. One of these rounds will be randomly selected by the computer and paid out.

In order to present a variety of decisions to you today, we collected decisions from participants B and C in advance, during previous experiment sessions. These participants were informed that we may select **some** of their decisions and match them with participants A, i.e. you. After their sessions participants B and C only received a fixed show-up payment. The additional payments, which participants B and C can earn through the decisions in the experiment, are still pending. These payments can only be calculated after you have made your decisions in step three and after one round has been selected randomly. Due to practicability reasons participants B and C will receive their final payments by post.

**Please note:** In order to simplify the experiment, you will make the **decision for the first step only once.** This means that you will decide at the beginning whether you will transfer your endowment of CHF 5 to the momentary participant C. This decision then remains valid for all of your participants C.

Thus, if you decide to transfer the CHF 5 to Participant C, your payoff consists of

the amount Participant C transfers back to you in the selected round

+ the additional CHF 5 received in step three

– any amount you decide to spend to reduce the payoff of Participant C in the selected round.

**Quiz**

Please answer the following questions to assess your understanding of the experiment.

**Question 1**

You and participant B both decide to transfer your CHF 5 to Participant C. This is multiplied by 4, resulting in a starting endowment for Participant C of CHF 20 from you and CHF 20 from Participant B. Participant C decides to send back 25% of what he received from you, resulting in CHF 5 for you and CHF 15 for Participant C. The computer decides to send back 25% of what Participant C received from Participant B, resulting in CHF 15 for Participant C and CHF 5 for Participant B. You receive an additional endowment of CHF 5 and decide to spend CHF 1 to reduce Participant C’s payoff. Your decision is “HIDDEN.”

Please calculate the following:

Your payoff:

Participant B’s payoff:

Participant C’s payoff:

Does participant C know whether you reduced his payoff (circle one)?

YES NO

**Question 2**

You and participant B both decide to transfer your CHF 5 to Participant C. This is multiplied by 4, resulting in a starting endowment for Participant C of CHF 20 from you and CHF 20 from Participant B. The computer decides to send back 50% of what Participant C received from you, resulting in CHF 10 for you and CHF 10 for Participant C. Participant C decides to send back 0% of what Participant C received from Participant B, resulting in CHF 20 for Participant C and CHF 0 for Participant B. You receive an additional endowment of CHF 5, and decide to spend CHF 5 to reduce Participant C’s payoff. Your decision is “OPEN.”

Please calculate the following:

Your payoff:

Participant B’s payoff:

Participant C’s payoff:

Does participant C know whether you reduced his payoff (circle one)?

YES NO

**Question 3**

You and participant B both decide to transfer your CHF 5 to Participant C. This is multiplied by 4, resulting in a starting endowment for Participant C of CHF 20 from you and CHF 20 from Participant B. The computer decides to send back 50% of what Participant C received from Participant B, resulting in CHF 10 for Participant B and CHF 10 for Participant C. The computer decides to send back 25% of what Participant C received from you, resulting in CHF 15 for Participant C and CHF 5 for you. You receive an additional endowment of CHF 5 and decide not to reduce Participant C’s payoff. Your decision is “HIDDEN.”

Please calculate the following:

Your payoff:

Participant B’s payoff:

Participant C’s payoff:

Does participant C know whether you reduced his payoff (circle one)?

YES NO

When you have finished the quiz, please raise your hand.

**Player B instructions**

We are seeking participants to make decisions that may be used in future experiments. By completing this brief questionnaire, you can possibly earn money in the future based on your decisions. Your decision might never be used; it might be used once; or it might be used more than once.

Please read the following instructions carefully. If you have any questions, please ask us.

There are two types of participants in this experiment, participants A and participants B. **You are a Participant A.** Participants B will take part in the experiment at a later date.

You will participate in a two-step experiment with a single participant B. In step one, you must decide whether you will **transfer money to Participant B** or if you will retain the money for yourself. In step two, Participant B will decide if he will transfer money back to you or if he will keep it for himself.

**Procedure for the two steps.** You will receive an endowment of **CHF 5** at the beginning of the first step.

You must decide in **step one** whether you want to transfer your endowment of CHF 5 to Participant B or if you will transfer nothing. If you transfer the CHF 5, it will be multiplied by 4, meaning that Participant B will receive a total endowment of CHF 20.

Participant B will be informed at the beginning of **step two** whether you transferred the CHF 5 to him. If you transferred the CHF 5, Participant B then decides whether he will transfer back 0%, 25%, or 50% of the total endowment to you. Note that Participant B makes his decision about the **percentage** of the endowment he will share with you. Participant B is informed that your transfer of CHF 5 will be multiplied by a factor between 2 and 6, but he does not know the size of the endowment when he makes his decision. Following Participant B’s decision, the size of the total endowment will be revealed. You will then receive exactly the amount of money Participant B transfers.

**Example:**

- You decide to transfer CHF 5 to Participant B.
- Participant B decides to share 50% of the endowment with you.
- The total endowment is revealed to be CHF 20 (initial transfer of CHF 5, multiplied by 4).
- You receive CHF 10 and Participant B receives CHF 10.

**Procedure of the entire experiment.** Today, you will make a single decision in step one: whether or not to transfer CHF 5 to a future participant B.

This decision may be used in a future experiment. If your decision is selected to be used in a future experiment, we will pay you based on your decision:

If you chose not to transfer the CHF 5 to Participant B, you will receive CHF 5.

If you chose to transfer the CHF 5 to a Participant B, we will present your decision to a Participant B, who is another participant in this experiment. After we have presented your decision to a Participant B, and Participant B has made his decision, we will calculate the payments for you and Participant B and send your payments by post.

**Please make your decision:**

Would you like to transfer CHF 5 to a future Participant B?

YES NO

***For payments to be transferred to you, please provide the following information:***

First name:

Last name:

E-Mail address:

(Important: this should be valid through 2013)

Postal address:

Street number/apartment number

City:

Postcode:

**Player T instructions**

We are seeking participants to make decisions that may be used in future experiments. By completing this brief questionnaire, you can possibly earn money in the future based on your decisions. Your decision might never be used; it might be used once; or it might be used more than once.

Please read the following instructions carefully. If you have any questions, please ask an experimenter.

There are three types of participants in this experiment, participants A, participants B, and participants C. **You are a Participant A.** Participants B and C will take part in the experiment at a later date.

You will participate in a three-step experiment with a single participant B and a single participant C. In step one, participants B and C must decide whether they will transfer money to you or if they will retain the money for themselves. In step two, if both participants B and C decided to transfer money to you, you will decide if you will transfer some money back to participants B and C, or if you will keep it for yourself. In step three, either participant C or participant B must again make a decision. There are various options in step three, which will be explained below. We will also describe the exact experiment procedure on the next pages.

**Procedure for the three steps.** Participants B and C will receive an endowment of **CHF 5** at the beginning of the first step.

They must decide in **step one** whether they want to transfer their endowment of CHF 5 to you or if they will transfer nothing. If they both transfer the CHF 5, it will be multiplied by a factor between 2 and 6, meaning that you will receive a total endowment of CHF 10–30 from both Participant B and Participant C.

There will be two decisions made in **step two**:

1. You will send back to Participant B 0%, 25%, or 50% of what you received from him and
2. You will send back to Participant C 0%, 25%, or 50% of what you received from him.

One of these decisions will be made by you, and the other will be made by the computer. You will not learn the decision made by the computer.

Note that you will make your decisions about the *percentage* of the endowment you will transfer back to Participants B and C. You will not know the size of the total endowment at the time you make your decision.

In **step three,** either Participant B or Participant C receives an additional CHF 5. He can then use this money to reduce your payoff. For every CHF 1 spent by Participant B or C, your payoff will be reduced by CHF 2.

At the end of step three, the final payoffs for all participants will be calculated. You will receive your final payment by post. Note that you will never learn the size of your starting endowment or the decision of the computer, so you won’t be able to know whether Participant B or C chose to reduce your payoff based on the size of your payoff alone. In some cases, you will not be informed whether Participant B or C chose to reduce your payoff. In other cases, you will be informed whether Participant B or C chose to reduce your payoff.

**Example:**

Participants B and C both decide to transfer their CHF 5 to you.

You decide to send back 25% of what you received from Participant B. The computer decides what percentage to send back to Participant C (but you do not learn the decision of the computer).

A few months later, you receive a payment of CHF 30 by post.

Note that this payment could be generated by more than one scenario. For example:

***Scenario 1:***

Participants B and C both decide to transfer their CHF 5 to you. This is multiplied by 4, resulting in a starting endowment of CHF 20 from Participant B and CHF 20 from Participant C. You decide to send back 25% of what you received from Participant B, resulting in CHF 5 for Participant B and CHF 15 for you. The computer decides to send back 25% of what you received from Participant C, resulting in CHF 15 for you and CHF 5 for Participant C. Participant B receives an additional endowment of CHF 5 and decides not to spend any money to reduce your payoff. Therefore the final payoffs for all participants are the following:

You (participant A): CHF 30

Participant B: CHF 10

Participant C: CHF 5

***Scenario 2:***

Participants B and C both decide to transfer their CHF 5 to you. This is multiplied by 5, resulting in a starting endowment of CHF 25 from Participant B and CHF 25 from Participant C. You decide to send back 25% of what you received from Participant B, resulting in CHF 6.25 for Participant B and CHF 18.75 for you. The computer decides to send back 25% of what you received from Participant C, resulting in CHF 18.75 for you and CHF 6.25 for Participant C. Participant B receives an additional endowment of CHF 5 and decides to spend CHF 3.75 to reduce your payoff by CHF 7.50. Therefore the final payoffs for all participants are the following:

You (participant A): CHF 30

Participant B: CHF 7.50

Participant C: CHF 6.25

Note that these are just two possible scenarios that could generate a final payoff for you of CHF 30.

**Procedure of the entire experiment.** Today, you will make 6 decisions. These decisions may be used in a future experiment. If your decision is selected to be used in a future experiment, we will pay you based on your decision and the decisions of Participants B and C.

You will make these 6 decisions *assuming that* Participants B and C both decided to transfer their CHF 5 to you. For each decision, you will be matched with different Participants B and C.

***Please make your decisions:***

1. Participants B and C both decided to transfer their CHF 5 to you. This amount is multiplied by a factor between 2 and 6. The computer will decide whether to send back 0%, 25%, or 50% of what you received from Participant B. Please make your decision about Participant C:

1. I would like to send back 0% of what I received from Participant C
2. I would like to send back 25% of what I received from Participant C
3. I would like to send back 50% of what I received from Participant C

2. Participants B and C both decided to transfer their CHF 5 to you. This amount is multiplied by a factor between 2 and 6. The computer will decide whether to send back 0%, 25%, or 50% of what you received from Participant B. Please make your decision about Participant C:

1. I would like to send back 0% of what I received from Participant C
2. I would like to send back 25% of what I received from Participant C
3. I would like to send back 50% of what I received from Participant C

3. Participants B and C both decided to transfer their CHF 5 to you. This amount is multiplied by a factor between 2 and 6. The computer will decide whether to send back 0%, 25%, or 50% of what you received from Participant B. Please make your decision about Participant C:

1. I would like to send back 0% of what I received from Participant C
2. I would like to send back 25% of what I received from Participant C
3. I would like to send back 50% of what I received from Participant C

4. Participants B and C both decided to transfer their CHF 5 to you. This amount is multiplied by a factor between 2 and 6. The computer will decide whether to send back 0%, 25%, or 50% of what you received from Participant B. Please make your decision about Participant C:

1. I would like to send back 0% of what I received from Participant C
2. I would like to send back 25% of what I received from Participant C
3. I would like to send back 50% of what I received from Participant C

5. Participants B and C both decided to transfer their CHF 5 to you. This amount is multiplied by a factor between 2 and 6. The computer will decide whether to send back 0%, 25%, or 50% of what you received from Participant B. Please make your decision about Participant C:

1. I would like to send back 0% of what I received from Participant C
2. I would like to send back 25% of what I received from Participant C
3. I would like to send back 50% of what I received from Participant C

6. Participants B and C both decided to transfer their CHF 5 to you. This amount is multiplied by a factor between 2 and 6. The computer will decide whether to send back 0%, 25%, or 50% of what you received from Participant B. Please make your decision about Participant C:

1. I would like to send back 0% of what I received from Participant C
2. I would like to send back 25% of what I received from Participant C
3. I would like to send back 50% of what I received from Participant C

***For payments to be transferred to you, please provide the following information:***

First name:

Last name:

E-Mail address:

(Important: this should be valid through 2013)

Postal address:

Street number/apartment number

City:

Postcode:

**Appendix 2**

**Example Decision Screens**

**
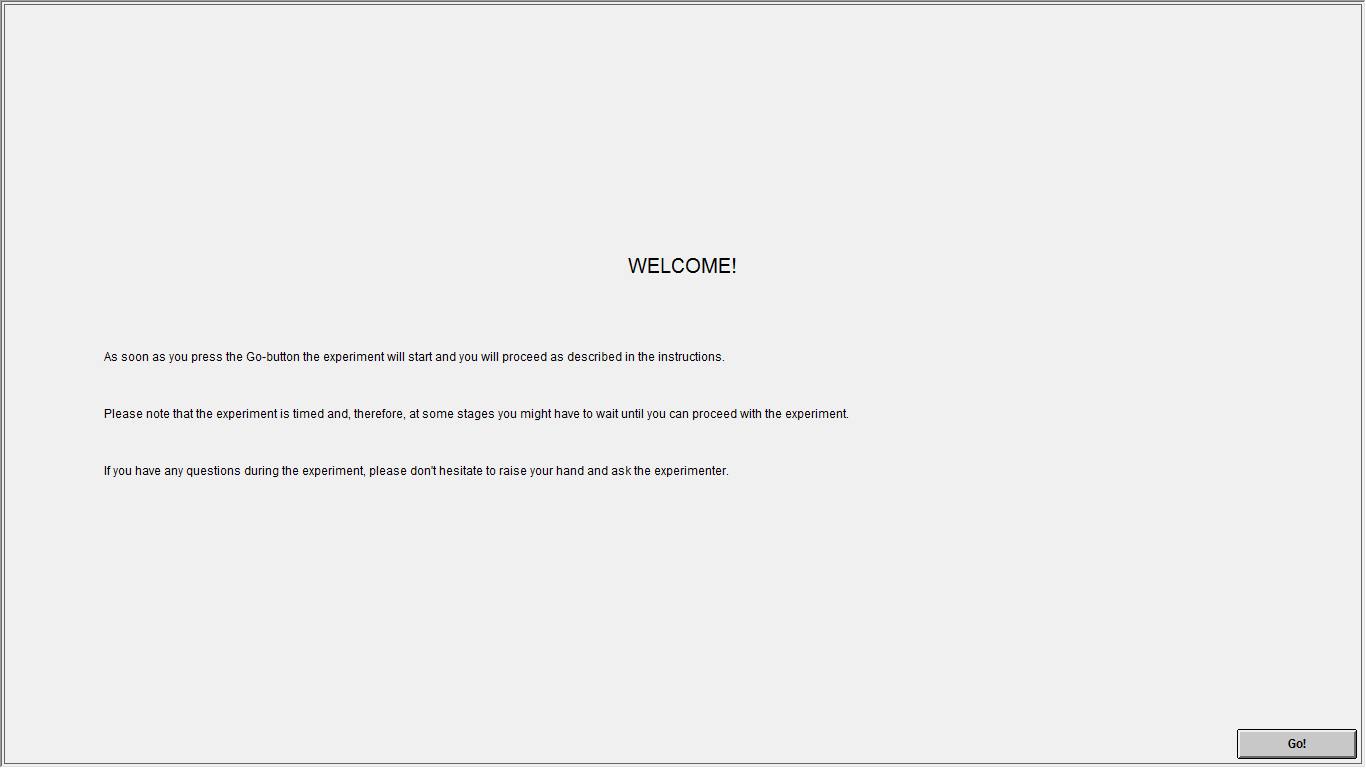
**

**
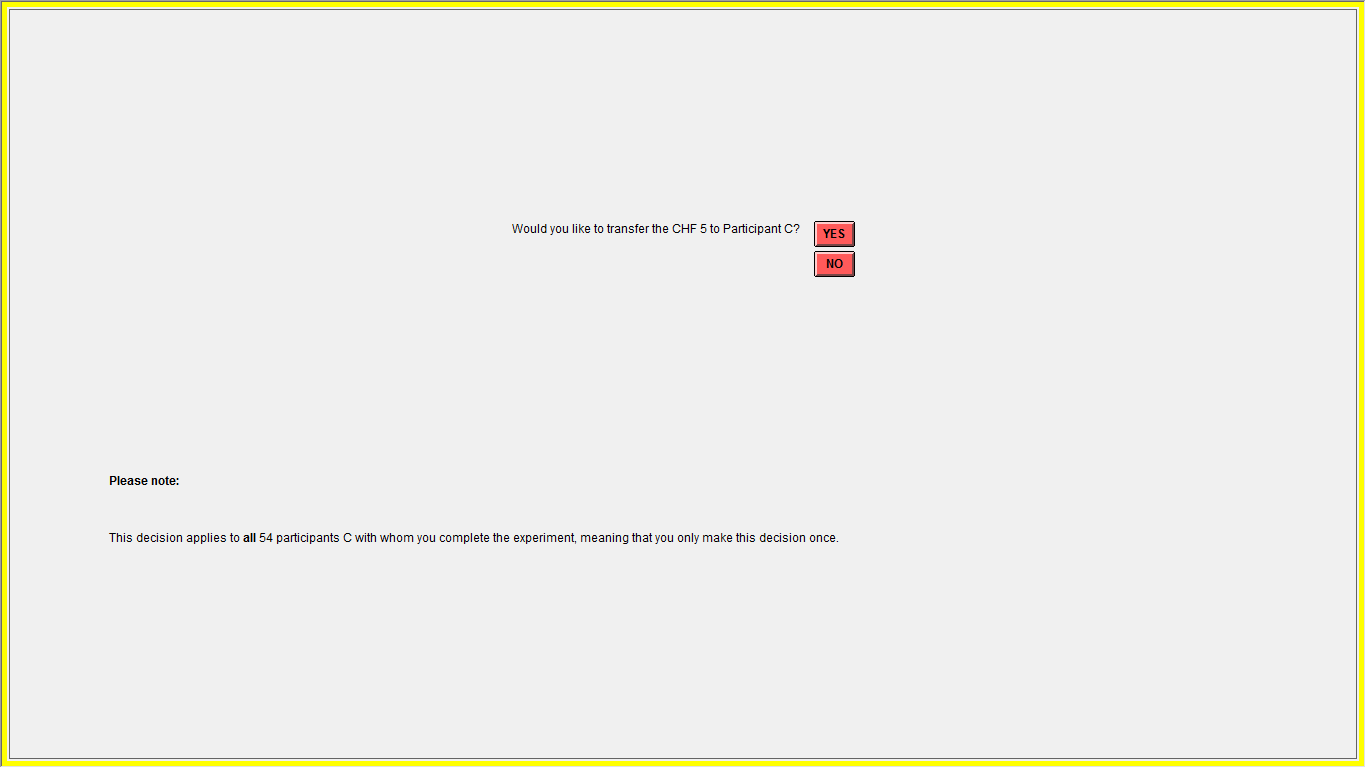
**

**
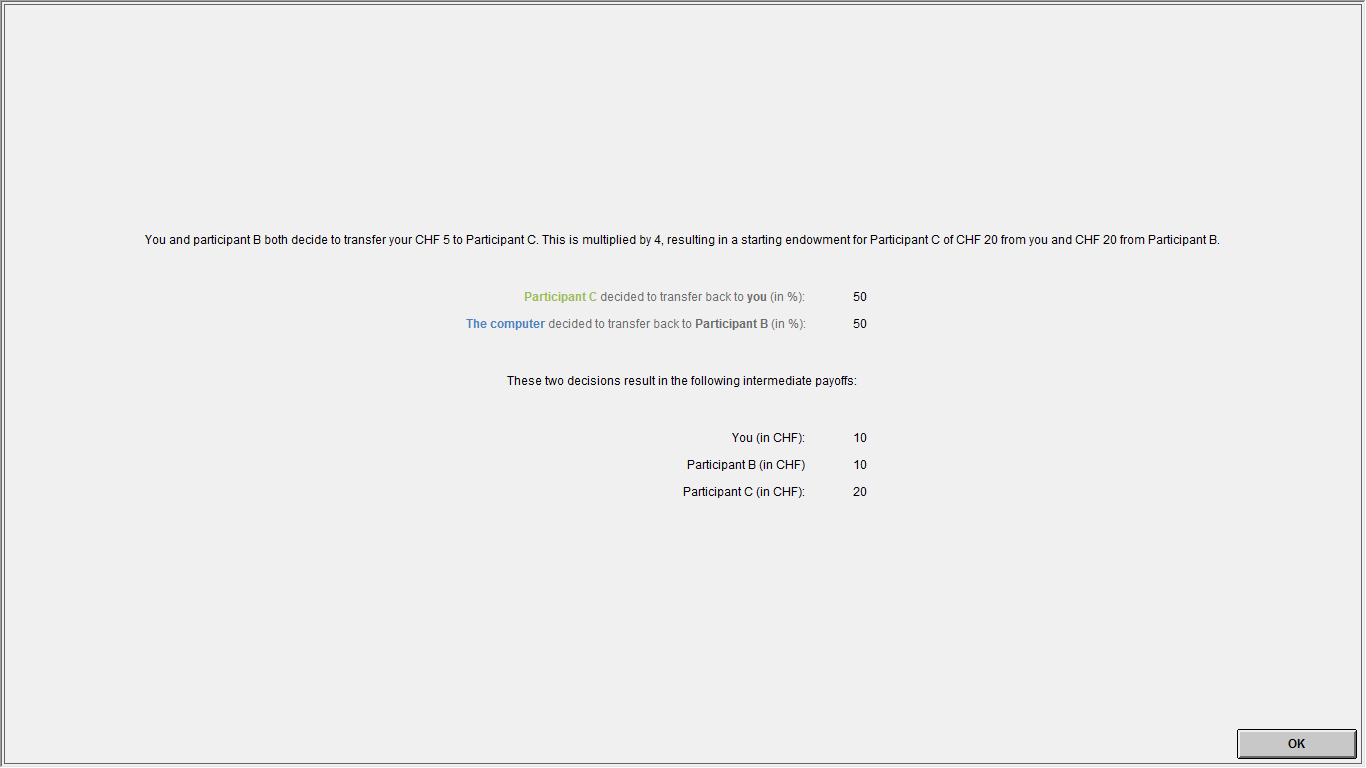
**

**
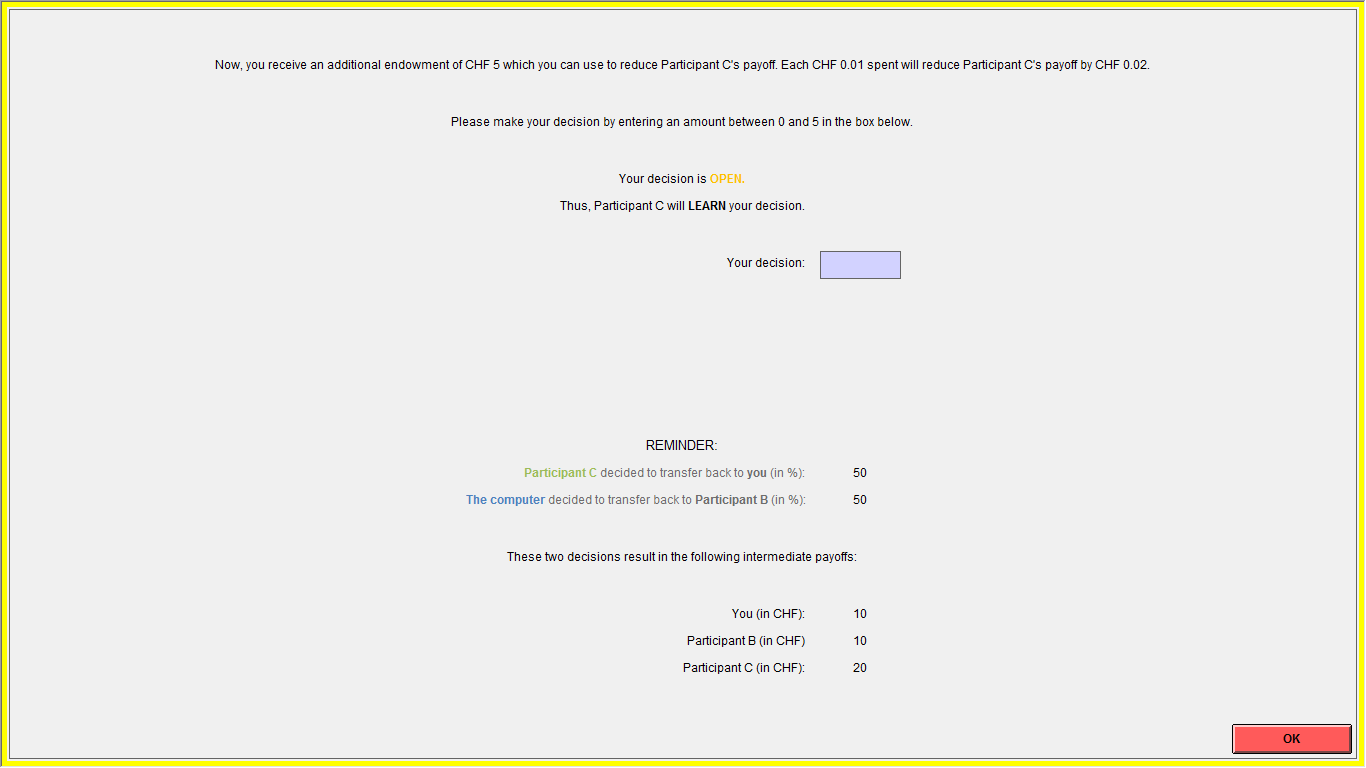
**


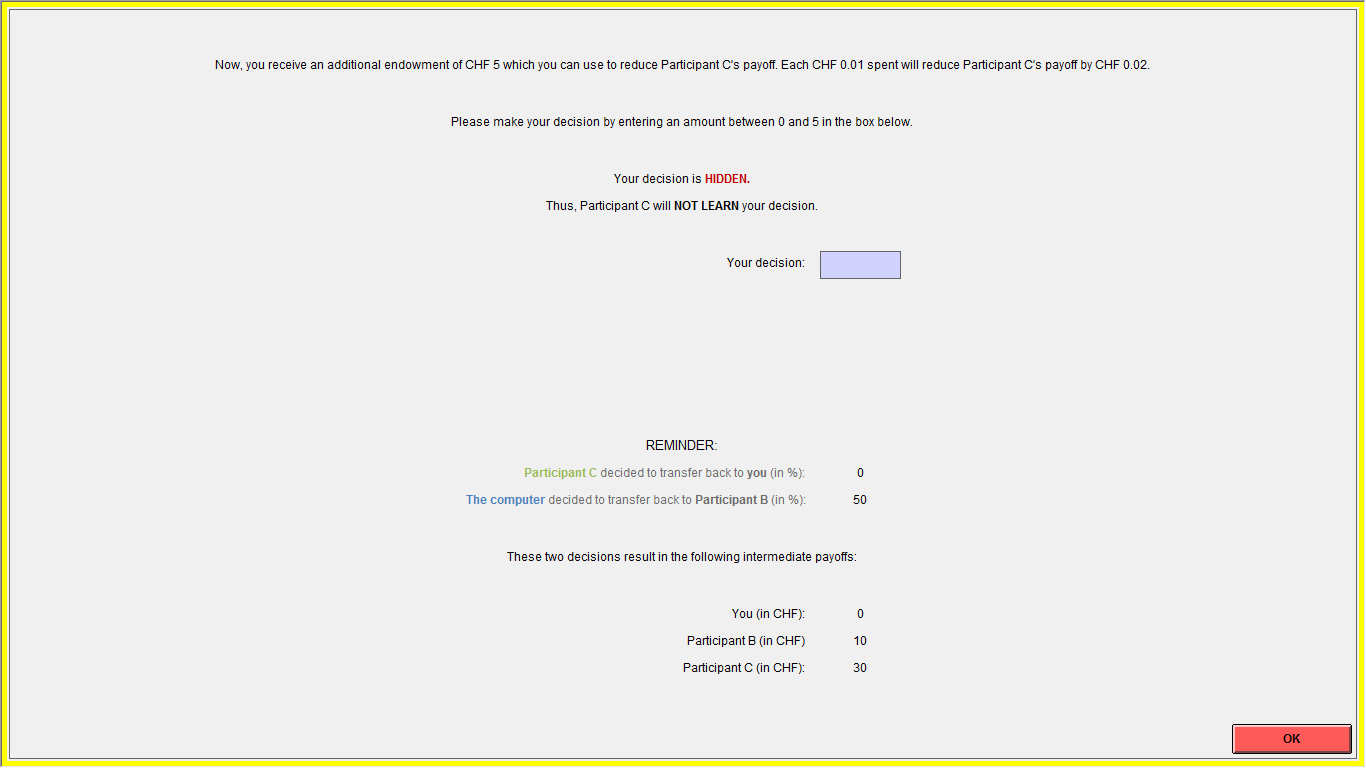


**
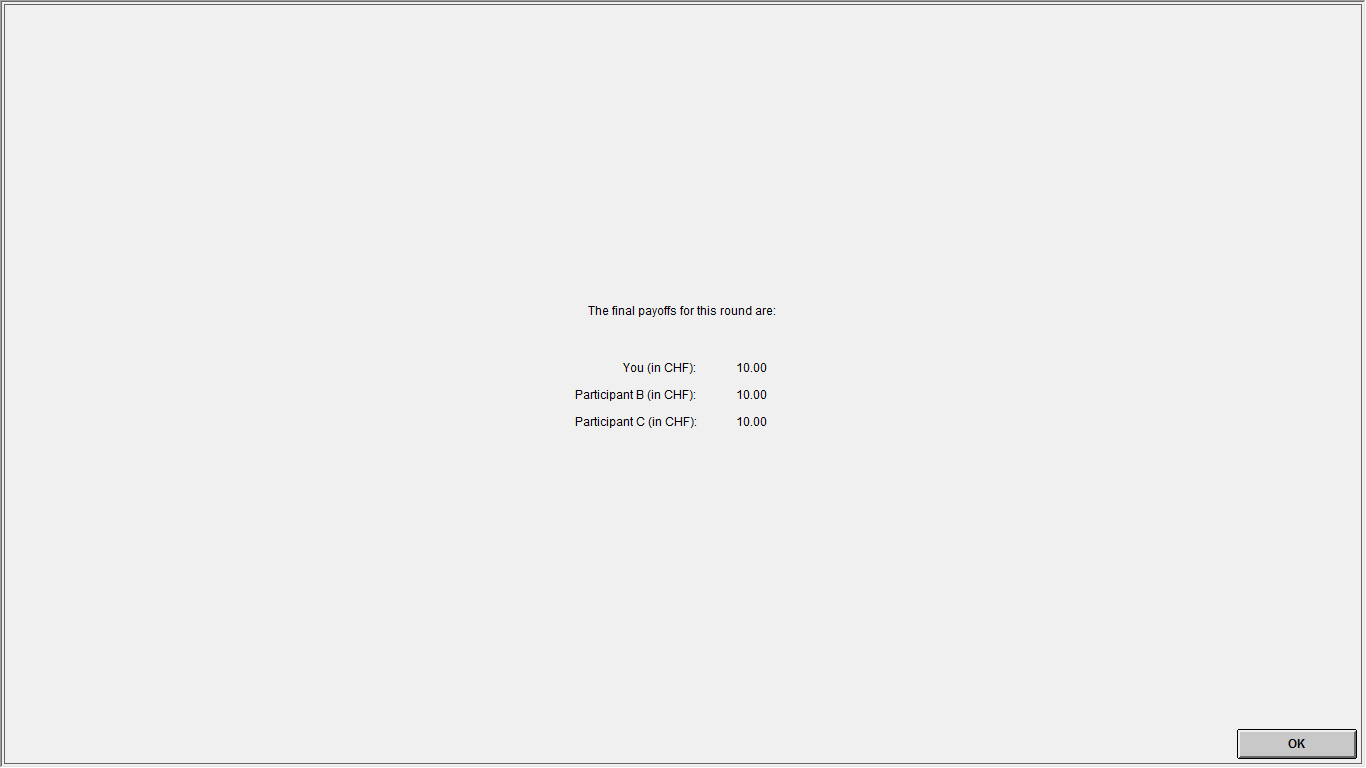
**

1. Note that payoffs are symmetric in i and j. [↑](#footnote-ref-1)
